# Evolution of the “fourth stage” of epidemiologic transition in people aged 80 years and over: population-based cohort study using electronic health records

**DOI:** 10.1186/s12963-017-0136-2

**Published:** 2017-05-12

**Authors:** Nisha C. Hazra, Martin Gulliford

**Affiliations:** 1 Department of Primary Care and Public Health Sciences, King’s College London, 3rd Floor Addison House, Guy’s Campus, London, SE1 1UL UK; 2 NIHR Biomedical Research Centre at Guy’s and St Thomas’ NHS Foundation Trust, Great Maze Pond, London, SE1 9RT UK

**Keywords:** Epidemiological transition, Incidence, Very old, Senior elderly, Chronic disease, Morbidity, Epidemiology, Primary care, UK

## Abstract

**Background:**

In the “fourth stage” of epidemiological transition, the distribution of non-communicable diseases is expected to shift to more advanced ages, but age-specific changes beyond 80 years of age have not been reported.

**Methods:**

This study aimed to evaluate demographic and health transitions in a population aged 80 years and over in the United Kingdom from 1990 to 2014, using primary care electronic health records. Epidemiological analysis of chronic morbidities and age-related impairments included a cohort of 299,495 participants, with stratified sampling by five-year age group up to 100 years and over. Cause-specific proportional hazards models were used to estimate hazard ratios for incidence rates over time.

**Results:**

Between 1990 and 2014, nonagenarians and centenarians increased as a proportion of the over-80 population, as did the male-to-female ratio among individuals aged 80 to 95 years. A lower risk of coronary heart disease (HR 0.54, 95% confidence interval [CI]: 0.50–0.58), stroke (0.83, 0.76–0.90) and chronic obstructive pulmonary disease (0.59, 0.54–0.64) was observed among 80–84 year-olds in 2010–2014 compared to 1995–1999. By contrast, the risk of type II diabetes (2.18, 1.96–2.42), cancer (1.52, 1.43–1.61), dementia (2.94, 2.70–3.21), cognitive impairment (5.57, 5.01–6.20), and musculoskeletal pain (1.26, 1.21–1.32) was greater in 2010–2014 compared to 1995–1999.

**Conclusions:**

Redistribution of the over-80 population to older ages, and declining age-specific incidence of cardiovascular and respiratory diseases in over-80s, are consistent with the “fourth stage” of epidemiologic transition, but increases in diabetes, cancer, and age-related impairment show new emerging epidemiological patterns in the senior elderly.

**Electronic supplementary material:**

The online version of this article (doi:10.1186/s12963-017-0136-2) contains supplementary material, which is available to authorized users.

## Background

The epidemiologic transition theory, proposed by Omran in 1971 [1], was developed from the demographic transition model [2] adding a more detailed consideration to causes of mortality [3]. The theory accounts for changes in age- and cause-specific mortality associated with development, generally characterized by a decline in mortality from infectious diseases and reproductive conditions, accompanied by the emergence of chronic non-communicable diseases as the most important causes of death [4]. Empirical studies from the United Kingdom [5–7] and other countries [3, 8] have supported the general outline of Omran’s theory, but have also identified some shortcomings. In middle- and low-income countries, the emergence of HIV-related mortality and the impact of war and civil conflict show that changes in mortality are not always consistent with the direction of theory [9]. In high-income countries, the epidemiologic transition theory as originally proposed did not account for the decline in non-communicable diseases that began to be observed soon after its publication. Olshansky and Ault [10] proposed a “fourth stage” of epidemiologic transition, “The Age of Delayed Degenerative Diseases,” in which declining age-specific mortality results in a gradual shift of non-communicable burden to older ages, with underlying causes of death showing little change overall. However, Modig et al. reported that case fatality following myocardial infarction (MI) has fallen steadily in Sweden in every year of age up to 100 since the mid-1990s [11]. This “fourth stage” of transition is associated with population aging and has assumed great importance as the senior elderly population, aged 80 years and older, has increased.

The epidemiologic transition theory is primarily concerned with changing patterns in mortality [12]. Morbidity, the incidence of diseases and health conditions, have generally been omitted from direct consideration, despite disease incidence being an important driving factor responsible for key outcomes described in the theory. There is a gap in our understanding with respect to age-specific changes in morbidity above the age of 80 years, with most studies analyzing all elderly together as a single group. The World Health Organization’s recent 2015 report on Ageing and Health [13] highlights the importance of characterizing aging processes as a crucial element of the health agenda. The present study therefore analyzes electronic health records (EHRs) from a large primary care database in the UK to describe demographic and epidemiological changes in a population of senior elderly aged 80 years and older between 1990 and 2014. EHRs have been identified as a valuable resource for research into the population effect of changing demography on the epidemiology of chronic illnesses [14]. Analyses specifically aimed to describe changes in the age and sex distribution of the senior elderly population and to relate these to changes in the incidence of chronic diseases and age-related impairments, as drivers of changes in mortality. The results are discussed in terms of the insight these provide into the progress of the “fourth stage” of epidemiologic transition in the UK.

## Methods

### Data source

Data were obtained from the Clinical Practice Research Datalink (CPRD), a nationally representative database of EHRs in the UK containing anonymized patient records for approximately 7% of the UK population [15–17]. The CPRD population has been shown to be representative of the UK population with respect to age, gender, and geographical distribution [15, 18]. Data collection occurs daily as a part of normal clinical care of patients registered with participating practices [15]. The frequency of data collection is determined by patient need and patients are included in the dataset from first contact until last contact with the practice. This population-based cohort study was approved through a protocol submitted to the Medicines and Healthcare Products Regulatory Agency (MHRA) Independent Scientific Advisory Committee (ISAC) for CPRD studies (Protocol No. 15_047).

### Demographic data and analysis

Demographic data were obtained from the March 2015 release of CPRD, including a complete listing of the start and end dates of CPRD records, year of birth, and date of death for all participants contributing data to CPRD. The proportion of senior elderly in the total CPRD population was determined based on individuals reaching the age of 80 years or greater, by sex and five-year period from 1990 to 2014, using mid-year counts. For each five-year period, the proportion of senior elderly in each five-year age-group, per 1,000 population, was estimated including changes over time in the past two decades and 95% confidence intervals calculated using the Poisson distribution. Mid-year counts were calculated by including individuals reaching the age of 80 years or greater, with an active record on 30 June during the year of interest. These were aggregated for individuals 80 years or over as the numerator, with mid-year counts for all ages as denominator. Sex ratios, as the number of men per 100 women, were estimated in each year for the populations aged ≥80, ≥85, ≥90, ≥95, and ≥100 years. Moving averages were used for the age groups ≥95 and ≥100 years in order to smooth the trend line due to smaller numbers. The age at death for individuals who died at the age of 80 years or over was plotted by year of death.

### Epidemiological data and analysis

For epidemiological analysis, a stratified random sample was selected from the list of all patients registered at CPRD general practices. The list of eligible participants was first divided into those who had reached their 80th, 85th, 90th, 95th, and 100th birthdays while registered with CPRD during the study period, 1st January 1990 to 31st December 2014. A random sample of up to a maximum of 50,000 men and 50,000 women was taken from each stratum, resulting in 299,495 participants (168,782 females and 130,713 males) aged 80 years and over, including 10,560 reaching 100 years or older.

Case definitions were developed for the outcomes of coronary heart disease (CHD) [19], stroke [20], type II diabetes mellitus (DM) [21], chronic obstructive pulmonary disease (COPD), and cancer (malignant neoplasms). Age-related impairments included dementia, cognitive and memory impairment, musculoskeletal (MSK) pain, falls, factures, and hearing impairment and visual impairment, as reported previously [22]. We estimated incidence rates for the onset of a new chronic condition or age-related impairment (Additional file 1), and used a cause-specific proportional hazards models to estimate hazard ratios [23, 24], reflecting changes over time. We acknowledge the potential importance of competing risks for future studies that might report on cumulative incidence of specific outcomes. Models were estimated separately for each five-year age-group including: 80–84 years, 85–89 years, 90–94 years, 95–99 years, and 100+ years. We contrasted two decades, divided into four five-year periods: 1995–1999, 2000–2004, 2005–2009, and 2010–2014. The years 1990–1994 were omitted in order to focus analysis on the most recent two decades. Multiple records were analyzed, based on successive age-groups and time periods for each participant. Hazard ratios (HRs) were adjusted for time and sex, and estimated in each age-group comparing each period to 1995–1999 as the reference period. The proportional hazards assumption was evaluated through estimation of Schoenfeld residuals. Analyses were conducted using Stata version 14.0.

## Results

The total CPRD registered population increased, following the recruitment of family practices, from 1.16 million in 1990–1994 to 4.77 million in 2005–2009 before declining to 4.45 million in 2010–2014 (Table 1). The number of individuals aged 80 years and older in CPRD increased from 53,008 (36,533 women, 16,475 men) in 1990–1994 to 226,479 (141,359 women, 85,119 men) in 2010–2014. The proportion of the total population aged ≥80 years increased from 45.7 per 1,000 (95% confidence interval [CI]: 45.3–46.1) in 1990–1994 to 50.9 per 1,000 (95% CI: 50.7–51.1) in 2010–2014; for women the increase was from 61.0 per 1,000 (95% CI: 61.2–62.5) to 63.0 per 1000 (95% CI: 62.7–63.3), and in men the increase was greater, from 28.9 per 1,000 (95% CI: 28.5–29.4) to 38.6 per 1,000 (95% CI: 38.4–38.9). While the proportion of men ≥80 years showed a consistent increase in successive periods, the proportion of women ≥80 years declined by 0.5 per 1,000 from 2005–2009 to 2010–2014 (Table 1).

**Table 1:** Proportion of CPRD population aged 80 years or older by five-year period and sex

Period	Total population		80+ years per thousand total population (95% CI)	Female population		80+ years per thousand total population (95% CI)	Male population		80+ years per thousand total population (95% CI)
	80+ years	All ages (millions)		80+ years	All ages (millions)		80+ years	All ages (millions)	
1990-1994	53,008	1.16	45.7 (45.3 to 46.1)	36,533	0.59	61.0 (61.2 to 62.5)	16,475	0.57	28.9 (28.5 to 29.4)
1995-1999	96,266	2.06	46.7 (46.4 to 47.0)	65,649	1.04	62.9 (62.4 to 63.4)	30,617	1.02	30.1 (29.8 to 30.4)
2000-2004	195,829	4.03	48.5 (48.3 to 48.8)	130,126	2.03	64.0 (63.7 to 64.4)	65,703	2.00	32.8 (32.6 to 33.1)
2005-2009	235,745	4.77	49.4 (49.2 to 49.6)	152,266	2.40	63.5 (63.2 to 63.8)	83.479	2.37	35.2 (35.0 to 35.4)
2010-2014	226,479	4.45	50.9 (50.7 to 51.1)	141,359	2.24	63.0 (62.7 to 63.3)	85,119	2.20	38.6 (38.4 to 38.9)

Figures are means of annual mid-year counts for each period, except where indicated

CI, confidence interval; CPRD, Clinical Practice Research Datalink

**Fig. 1:**
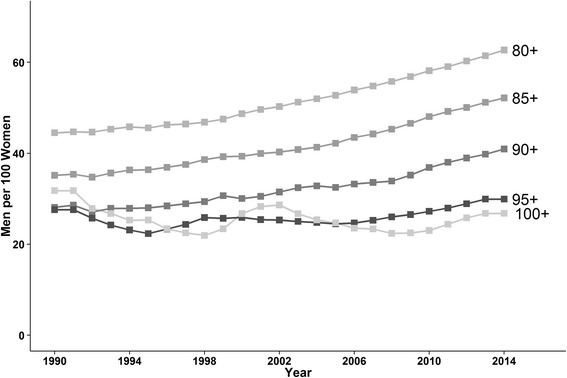
Sex ratios of the number of men per 100 women between 1990 and 2014

**Fig. 2:**
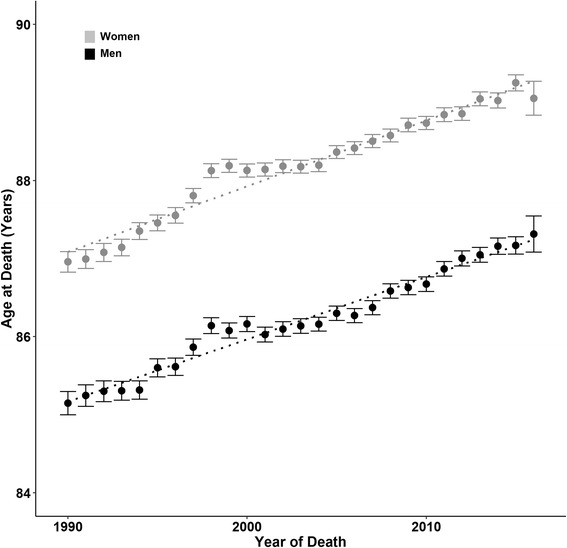
Mean age at death for senior elderly by year of death. Legend: *Grey*, Women; *Black*, Men; Bars are 95% confidence intervals

As expected, each successive five-year age-group over 80 years represented a decreasing proportion of the total senior elderly population (Table 2). However, the oldest age groups generally showed the largest relative increases over time. The proportion of women aged 80–84 years decreased from 36.5% of the total over-80 population to 29.0% between 1990–1994 and 2010–2014. Men aged 90–94 years and women aged 100 years and older were the fastest growing groups, as proportions of all over-80s. Sex difference in the numbers of senior elderly has declined over the past two decades (Fig. 1); the ratio of men per 100 women aged 80 years and older increased over the study period from 44.5 men per 100 women in 1990 to 62.7 men per 100 women in 2014. In the 85-and-over age-group, the sex gap narrowed from 35.1 men per 100 women to 52.2 per 100, and in the 90-and-over age-group from 28.1 to 40.9 men per 100 women. A lesser change over time is observed for the group aged 95 years or greater. Since 1990, mean age at death among the senior elderly increased by two years; from 87 years to 89 years in women, and from 85 to 87 years in men (Fig. 2).

**Table 2:** Proportion of the population aged 80 years and over by age-group and sex

	80-84 years		85-89		90-94		95-99		100+		Total men & women 80+
MEN											
1990-1994	51,820	(19.6)	22,429	(8.5)	6,502	(2.5)	1,317	(0.50)	309	(0.12)	265,042
1995-1999	89,043	(18.5)	46,168	(9.6)	14,270	(3.0)	3,150	(0.65)	452	(0.09)	481,328
2000-2004	198,396	(20.3)	89,394	(9.1)	32,699	(3.3)	6,642	(0.68)	1,385	(0.14)	979,146
2005-2009	241,232	(20.5)	126,346	(10.7)	39,629	(3.4)	8,693	(0.74)	1,497	(0.13)	1,178,725
2010-2014	236,290	(20.9)	129,861	(11.5)	48,552	(4.3)	9,176	(0.81)	1,717	(0.15)	1,132,393
Change relative to 1990-1994		+6.7%		+35.5%		+74.8%		+63.1		+30.1	
WOMEN											
1990-1994	96,683	(36.5)	56,824	(21.4)	22,761	(8.6)	5,313	(2.0)	1,084	(0.41)	265,042
1995-1999	159,566	(33.2)	107,675	(22.4)	46,473	(9.7)	12,545	(2.6)	1,986	(0.41)	481,328
2000-2004	328,522	(33.6)	193,143	(19.7)	97,149	(9.9)	26,792	(2.7)	5,024	(0.51)	979,146
2005-2009	364,524	(30.9)	248,815	(21.1)	107,778	(9.1)	33,685	(2.9)	6,526	(0.55)	1,178,725
2010-2014	328,498	(29.0)	225,332	(19.9)	115,227	(10.2)	31,016	(2.7)	6,724	(0.59)	1,132,393
Change relative to 1990-1994		-20.5%		-7.2%		+18.5%		+36.6%		+45.2%	

Figures are sum of mid-year counts (% of total population aged 80 years and over)

**Fig. 3:**
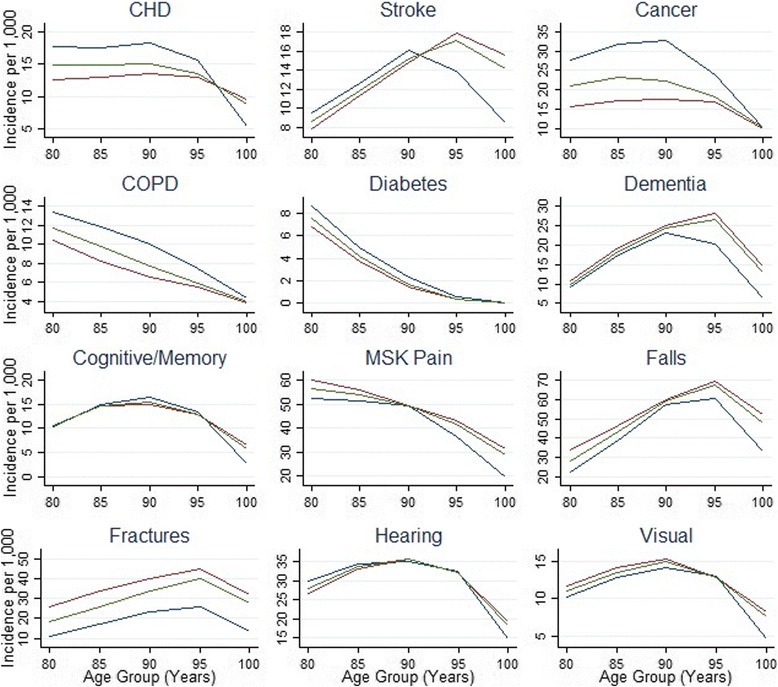
Sex-specific incidence rates of morbidities and impairments by age. Legend: *Blue*, males; *Red*, females; *Green*, overall

Changes over time in the risk of new chronic conditions are presented in Table 3, with the total number of events (Additional file 1) and overall incidence rates by age-group presented in Fig. 3. The relative hazard for CHD declined over time; at 80–84 years, CHD incidence was 46% lower (HR 0.54, 95% CI: 0.50–0.58) in 2010–2014 compared to 1995–1999 (Table 3). This decline was consistently observed in each age-group, with the exception of centenarians presenting a higher relative hazard for CHD in the most recent five-year period. This increased relative hazard might suggest a cohort effect but the data were not consistent, with a lower relative hazard being observed in earlier years. This may be a result of smaller numbers in this oldest age-group given the multiple comparisons being undertaken. The relative hazard for stroke at 90–94 years was 41% lower (HR 0.59, 95% CI: 0.54–0.65) in 2010–2014 compared to baseline. A decrease in stroke over time was observed in all five-year age-groups over 80 years. The relative hazard for COPD was 76% lower (HR 0.24, 95% CI: 0.18–0.30) in 2010–14 among those aged 95–99 years. By contrast, relative hazards for cancer and diabetes increased over the study period. In 2010–2014, cancer risk was 38% higher for 90–94 year olds and 69% higher for centenarians compared to 1995–1999. All age groups showed a similar successive risk increase for cancer with each five-year period. Diabetes risk increased by 118% for 80–84 year olds, and 451% for 85–89 year olds over the study period, but there were no new reported cases of diabetes over the age of 90 years.

**Table 3:** Age-specific changes in incidence of selected chronic conditions, 1995 to 2014

Condition	Age group (years)	Hazard ratio^a^ (95% CI)			
		1995-1999	2000-2004	2005-2009	2010-2014
CHD	80-84	Ref.	0.93 (0.88 to 0.98)	0.63 (0.59 to 0.67)	0.54 (0.50 to 0.58)
	85-89	Ref.	0.88 (0.82 to 0.94)	0.62 (0.58 to 0.66)	0.55 (0.51 to 0.60)
	90-94	Ref.	0.90 (0.82 to 0.99)	0.67 (0.61 to 0.74)	0.59 (0.67 to 0.76)
	95-99	Ref.	0.92 (0.78 to 1.10)	0.67 (0.56 to 0.79)	0.70 (0.59 to 0.84)
	100+	Ref.	0.78 (0.46 to 1.33)	0.56 (0.32 to 0.96)	1.96 (1.17 to 3.30)
Stroke	80-84	Ref.	0.95 (0.88 to 1.02)	0.80 (0.74 to 0.87)	0.83 (0.76 to 0.90)
	85-89	Ref.	0.83 (0.77 to 0.89)	0.68 (0.63 to 0.73)	0.71 (0.66 to 0.77)
	90-94	Ref.	0.78 (0.71 to 0.84)	0.63 (0.58 to 0.69)	0.59 (0.54 to 0.65)
	95-99	Ref.	0.79 (0.69 to 0.91)	0.56 (0.48 to 0.64)	0.53 (0.46 to 0.62)
	100+	Ref.	0.58 (0.40 to 0.85)	0.51 (0.35 to 0.74)	0.44 (0.30 to 0.65)
COPD	80-84	Ref.	0.66 (0.62 to 0.71)	0.63 (0.59 to 0.68)	0.59 (0.54 to 0.64)
	85-89	Ref.	0.57 (0.52 to 0.62)	0.52 (0.48 to 0.56)	0.47 (0.43 to 0.52)
	90-94	Ref.	0.47 (0.42 to 0.53)	0.35 (0.31 to 0.40)	0.36 (0.32 to 0.41)
	95-99	Ref.	0.40 (0.32 to 0.51)	0.28 (0.22 to 0.35)	0.24 (0.18 to 0.30)
	100+	Ref.	0.44 (0.23 to 0.86)	0.31 (0.16 to 0.62)	0.15 (0.07 to 0.35)
Cancer	80-84	Ref.	1.08 (1.02 to 1.15)	1.31 (1.24 to 1.39)	1.52 (1.43 to 1.61)
	85-89	Ref.	1.07 (1.00 to 1.14)	1.36 (1.28 to 1.45)	1.45 (1.36 to 1.54)
	90-94	Ref.	1.15 (1.05 to 1.26)	1.33 (1.22 to 1.45)	1.38 (1.26 to 1.50)
	95-99	Ref.	1.08 (0.91 to 1.28)	1.20 (1.02 to 1.42)	1.44 (1.22 to 1.70)
	100+	Ref.	0.87 (0.46 to 1.63)	1.17 (0.65 to 2.12)	1.69 (0.95 to 3.00)
Diabetes mellitus^b^	80-84	Ref.	1.62 (1.46 to 1.78)	2.02 (1.83 to 2.23)	2.18 (1.96 to 2.42)
	85-89	Ref.	2.45 (2.01 to 2.99)	3.65 (3.02 to 4.41)	5.41 (4.47 to 6.53)

*CI* confidence interval

^a^Adjusted for time and sex

^b^No incident diabetes events over 90 years of age

Incidence of age-related impairments has generally increased over time (Table 4) across all age-groups. New diagnoses of dementia increased by almost two-fold since 1995–1999 among individuals aged 80–84 years (HR 2.94, 95% CI: 2.70–3.21), while newly recorded cognitive impairment and memory problems were 4–5 times higher in 2010–2014 compared to 1995–1999 among those aged 80–84 years (HR 5.57, 95% CI: 5.01–6.20), 90–94 years (HR 6.30, 95% CI: 5.40–7.34) and 95–99 years (HR 6.62, 95% CI: 4.89–8.95). MSK pain, hearing impairment, falls, and fractures increased by approximately 30–40% in 80–84 year olds, with larger increases in older age-groups. Risk of falls and fractures among centenarians increased by 50% (HR 1.50, 95% CI: 1.12–2.02) and 57% (HR 1.57, 95% CI: 1.09–2.27), respectively. Figure 3 illustrates incidence rates across the whole study period by age-group. Incidence of the majority of impairments increased with age, declining beyond 95 years and in centenarians. In contrast, the majority of chronic conditions declined with age, except for stroke which increased from 80 to 95 years, and presented lower rates among centenarians.

**Table 4:** Age-specific changes in incidence of selected age-related impairments over the age of 80 years, 1995 to 2014

Condition	Age group (years)	Hazard ratio^a^ (95% CI)			
		1995-1999	2000-2004	2005-2009	2010-2014
Dementia	80-84	Ref.	1.28 (1.17 to 1.39)	1.85 (1.70 to 2.01)	2.94 (2.70 to 3.21)
	85-89	Ref.	1.21 (1.12 to 1.30)	1.55 (1.44 to 1.66)	2.40 (2.23 to 2.57)
	90-94	Ref.	1.16 (1.06 to 1.26)	1.50 (1.37 to 1.63)	2.03 (1.87 to 2.20)
	95-99	Ref.	1.32 (1.12 to 1.55)	1.76 (1.51 to 2.04)	2.43 (2.09 to 2.82)
	100+	Ref.	1.30 (0.70 to 2.42)	2.18 (1.21 to 3.90)	2.52 (1.41 to 4.50)
Cognitive/memory	80-84	Ref.	2.29 (2.05 to 2.55)	3.61 (3.25 to 4.01)	5.57 (5.01 to 6.20)
	85-89	Ref.	2.36 (2.11 to 2.63)	3.84 (3.46 to 4.27)	5.64 (5.08 to 6.27)
	90-94	Ref.	2.53 (2.16 to 2.98)	4.25 (3.64 to 4.96)	6.30 (5.40 to 7.34)
	95-99	Ref.	2.49 (1.81 to 3.42)	4.31 (3.17 to 5.84)	6.62 (4.89 to 8.95)
	100+	Ref.	2.28 (0.67 to 7.77)	3.96 (1.22 to 12.8)	6.02 (1.88 to 19.3)
MSK pain	80-84	Ref.	1.06 (1.02 to 1.10)	1.30 (1.25 to 1.35)	1.26 (1.21 to 1.32)
	85-89	Ref.	1.06 (1.01 to 1.11)	1.29 (1.23 to 1.35)	1.22 (1.16 to 1.28)
	90-94	Ref.	1.00 (0.93 to 1.06)	1.25 (1.17 to 1.33)	1.18 (1.11 to 1.26)
	95-99	Ref.	1.00 (0.88 to 1.14)	1.33 (1.18 to 1.50)	1.32 (1.16 to 1.49)
	100+	Ref.	0.83 (0.55 to 1.25)	1.45 (1.00 to 2.10)	1.63 (1.12 to 2.37)
Falls	80-84	Ref.	1.08 (1.03 to 1.13)	1.18 (1.13 to 1.24)	1.26 (1.19 to 1.32)
	85-89	Ref.	1.05 (1.00 to 1.10)	1.11 (1.06 to 1.16)	1.18 (1.13 to 1.23)
	90-94	Ref.	0.96 (0.91 to 1.02)	1.11 (1.05 to 1.17)	1.13 (1.07 to 1.19)
	95-99	Ref.	1.03 (0.94 to 1.14)	1.21 (1.11 to 1.33)	1.25 (1.14 to 1.37)
	100+	Ref.	1.10 (0.81 to 1.49)	1.33 (0.99 to 1.79)	1.50 (1.12 to 2.02)
Fractures	80-84	Ref.	0.94 (0.89 to 1.00)	1.18 (1.12 to 1.26)	1.38 (1.30 to 1.47)
	85-89	Ref.	0.96 (0.91 to 1.02)	1.20 (1.13 to 1.27)	1.49 (1.41 to 1.58)
	90-94	Ref.	0.97 (0.90 to 1.05)	1.19 (1.11 to 1.28)	1.42 (1.32 to 1.53)
	95-99	Ref.	1.11 (0.97 to 1.27)	1.41 (1.24 to 1.60)	1.61 (1.42 to 1.83)
	100+	Ref.	0.95 (0.64 to 1.41)	1.32 (0.91 to 1.92)	1.57 (1.09 to 2.27)
Hearing impairment	80-84	Ref.	1.14 (1.08 to 1.19)	1.26 (1.20 to 1.32)	1.28 (1.22 to 1.35)
	85-89	Ref.	1.21 (1.15 to 1.28)	1.34 (1.27 to 1.41)	1.32 (1.25 to 1.39)
	90-94	Ref.	1.22 (1.13 to 1.32)	1.40 (1.30 to 1.51)	1.43 (1.32 to 1.54)
	95-99	Ref.	1.40 (1.20 to 1.62)	1.65 (1.43 to 1.91)	1.81 (1.56 to 2.10)
	100+	Ref.	0.85 (0.51 to 1.41)	1.41 (0.88 to 2.25)	1.75 (1.10 to 2.78)
Visual impairment	80-84	Ref.	0.77 (0.72 to 0.82)	0.62 (0.58 to 0.66)	0.50 (0.46 to 0.54)
	85-89	Ref.	0.85 (0.80 to 0.91)	0.69 (0.65 to 0.74)	0.54 (0.50 to 0.59)
	90-94	Ref.	0.92 (0.84 to 1.01)	0.86 (0.79 to 0.95)	0.67 (0.61 to 0.74)
	95-99	Ref.	1.03 (0.85 to 1.25)	1.00 (0.83 to 1.20)	0.88 (0.73 to 1.07)
	100+	Ref.	0.76 (0.44 to 1.31)	0.74 (0.44 to 1.25)	0.51 (0.29 to 0.90)

*CI* confidence interval

^a^Adjusted for time and sex

## Discussion

Our data illustrate that the population aged 80 years and older is growing rapidly, with the most rapid growth, in relative terms, being among men aged 90 to 94 years and women aged 100 years or greater. The sex gap in numbers of senior elderly is reducing while the mean age at death is increasing. Our study also presents new morbidity estimates for cause-specific hazards in the senior elderly, which are among the first age-stratified estimates reported for men and women aged 80 to 100 years or greater. These results offer support to the proposed shift in mortality from non-communicable diseases to advanced ages, consistent with the “fourth stage” of epidemiologic transition or “The Age of Delayed Degenerative Diseases.” However, this empirical data also indicate that new and important epidemiological patterns are emerging in the senior elderly. While age-specific incidence for cardiovascular and respiratory diseases is declining, rates of diabetes and cancer are increasing substantially. In addition, a range of age-related impairments are increasingly occurring and rising in importance at the oldest ages, including dementia, cognitive impairment, and falls. This is consistent with the emergence of “frailty,” characterized by the accumulation of functional deficits [25], as a key epidemiologic finding in the latest stage of epidemiologic transition.

### Comparison with existing literature

While the decline in cardiovascular diseases (CVDs) is well-described, evidence for the extremes of age is sparse, but some studies have suggested declining incidence and mortality from CHD in adults aged more than 75 years [26, 27]. There is also evidence of a decline in CVD risk in Sweden, including both MI and stroke, for the period between 1994 to 2010 observed beyond 85 years, with the rate of improvement plateauing with age [11]. This is consistent with our findings for CHD, but not for stroke. Stroke incidence in the UK is reported to have declined by 42% between 1999 and 2008 in individuals aged 80 years or greater [28]. We similarly found declines in stroke incidence over time, but also report these decreases among the senior elderly becoming larger in magnitude with each increasing age-group up to 100+ years; a 17% decline (HR 0.83, 95% CI: 0.76–0.90) in incidence at 80–84 years and a 56% decline (HR 0.44, 95% CI: 0.30–0.65) in centenarians between 1995–1999 and 2010–2014. Declining trends in classic cardiovascular risk factors in recent years, including reductions in smoking, high blood pressure, and cholesterol levels [29–31], may be contributing to the observed reductions in cardiovascular and respiratory diseases in our study. The use of anti-hypertensive medications has also increased over the past two decades [31], consistent with our observed declines in CHD and other reports on reductions in CHD risk [32] and CHD mortality [26]. In contrast, recent research in the UK report mortality rising in many places among pensioners over 85, possibly linked to increasing austerity measures [33], which may be explained by a shift in morbidity and mortality away from CVD and towards other types of fatal chronic conditions and impairments. Changes in COPD incidence rates over time have not been reported in the UK elderly and reports from the United States give conflicting results [34, 35]. Among adults in the UK, a peak in age-sex standardized COPD incidence was reported in 2004, suggesting we may have reached a possible summit of COPD incidence and prevalence in England overall [36], however we report consistent declines in COPD incidence since 1995–1999 in all age-groups of senior elderly. Diabetes prevalence has increased worldwide [37], as has the age-adjusted incidence of diabetes in the US [38] and the distribution of obesity as a key risk factor for diabetes [39, 40]. Our findings reveal that increases in diabetes risk extend to all age-groups of senior elderly beyond 80 years of age. Increasing risk of cancer in all age-groups of our cohort is supported by reports from Cancer Research UK for individuals aged 75 and over [41], as well as increasing adiposity indicators and obesity [30] reported for the UK in recent decades.

The occurrence of new age-related impairments in our cohort, particularly degenerative mental health conditions, have risen steadily since the mid-90s. The reasons for this change remain poorly understood, but may be resulting from increased longevity associated with lower risk of cardiovascular mortality [35]. Age-specific dementia prevalence in the UK among over-65 s was previously reported to have fallen between 1989 and 2008 [42], contrasting to our findings showing an increasing frequency of new dementia and cognitive impairment diagnoses in over-80s over the past two decades. These opposing trends may be indicative of a shift in dementia towards more extreme ages, with falling prevalence in younger elderly and increasing incidence in the senior elderly. Our observed increases in the risk of hearing impairment may be explained by better diagnosis in primary care over time or improved access to treatment resulting in increased recording of diagnostic codes in CPRD. It is also possible that there have been improvements in the delivery of care, which could explain the observed declines in visual impairment among over-80s as a result of improvements in diabetic retinopathy screening and increased rates of cataract extractions. At older age groups, the incidence of falls and fractures presented large increases over time, with larger increases in fractures compared to falls. Increasing polypharmacy in older age, with a greater likelihood of drug interactions or inappropriate prescribing may sometimes contribute to iatrogenic risks such as falls [31, 43, 44]. This increase may also be attributed to increasing numbers of older people living alone in the UK and not making appropriate adaptations to their homes, as recently reported by the International Longevity Centre (ILC-UK) [45]. Recent increases in age-related impairments in our cohort reveal an important emerging characteristic of the current stage of epidemiologic transition.

### Strengths and limitations

This study has the strengths of a large nationally representative data resource, enabling us to analyze longitudinal demographic data for up to 7% of the UK population. Use of primary care EHRs facilitated analysis of longitudinal and age-specific estimates for a wide range of chronic morbidities and age-related impairments in a large sample. Approximately 98% of the UK population will be registered with primary care [46], ensuring our results are population-based. We acknowledge that misclassification of birth year may occur, especially for people born longer ago, due to poor recording practices at time of birth or if birth date is reported by proxies. The analysis used clinical records for health conditions and these may be affected by misclassification including over- or under-recording, or changes in the depth and quality of coding, which may vary over time. In general, diagnoses recorded into EHRs have high predictive value [47], but we acknowledge that diagnoses may be less reliable at older ages if, for example, fewer investigations are performed. Recording of age-related conditions such as dementia may be underestimated, however our data should provide reliable figures for relative changes over time. There may also be changes over recent years in the use of particular diagnostic labels. While these reservations suggest the need for further epidemiological studies in very old people, prospective cohort studies may suffer from different limitations including biases from differential selection, participation, and attrition. These may be minimized through the use of EHRs for cohort selection and follow-up.

## Conclusions

Empirical evidence reveals that the “fourth stage” of epidemiologic transition is more nuanced than originally proposed. At the oldest ages, populations exhibit important epidemiologic changes that are not yet well-characterized beyond 80 years. We recommend that, where data permits, the practice of reporting on “the elderly” as one group must change in order to more fully understand the distribution of disease and disability in different subgroups of the over-80 population. This will be a vital component in producing better evidence to improve the health and care of the senior elderly, and to help inform a shift in thinking from “chronic disease prevention” to “healthy aging” that is important at the oldest ages. Greater acknowledgement among policymakers and clinicians of the observed growth in multi-morbidity and shift away from the presently existing single-disease framework in health care [48] will be crucial in the coming years to better manage the health care needs of an aging population. Improved public health interventions are also required to reduce levels of obesity in the middle-aged and elderly populations in order to facilitate the prevention of new diabetes and cancer diagnoses in older age. However, more evidence is also needed to inform the prevention of accumulating functional deficits and age-related impairments.

## Additional file

Additional file 1: Table S1. Incidence rates of chronic morbidities by age group, 1995-2014. **Table S2.** Incidence rates of age-related impairments by age group, 1995-2014. **Table S3.** Incidence rates for chronic morbidities by ten-year period. **Table S4.** Incidence rates for age-related impairments by ten-year period. (DOCX 35 kb)

## Abbreviations

UK: United Kingdom
EHR: Electronic health record
CPRD: Clinical Practice Research Datalink
MHRA: Medicines and Healthcare Products Regulatory Agency
ISAC: Independent Scientific Advisory Committee
CHD: Coronary heart disease
DM: Diabetes mellitus
COPD: Chronic obstructive pulmonary disease
MSK: Musculoskeletal
HR: Hazard ratio
US: United States
