# Erratum to: Evolution of the “fourth stage” of epidemiologic transition in people aged 80 years and over: population-based cohort study using electronic health records

**DOI:** 10.1186/s12963-017-0147-z

**Published:** 2017-08-04

**Authors:** Nisha C. Hazra, Martin Gulliford

**Affiliations:** 1 Department of Primary Care and Public Health Sciences, King’s College London, 3rd Floor Addison House, Guy’s Campus, London, SE1 1UL UK; 2 NIHR Biomedical Research Centre at Guy’s and St Thomas’ NHS Foundation Trust, Great Maze Pond, London, SE1 9RT UK

## Erratum

Additional file 1 shown in the original article [1] is the incorrect version. The correct version is shown below:

## Additional file

Additional file 1: Table S1. Incidence rates^1^ of chronic morbidities by age group, 1995–2014. (DOCX 32 kb)
