# Supplementary material for: Evolution of the “fourth stage” of epidemiologic transition in people aged 80 years and over: population-based cohort study using electronic health records
Source: Popul Health Metr. 2017 May 12;15:18. doi: 10.1186/s12963-017-0136-2 (PMC5429583; doi:10.1186/s12963-017-0136-2)
Supplement: Additional file 1: Table S1. — Incidence rates of chronic morbidities by age group, 1995-2014. Table S2. Incidence rates of age-related impairments by age group, 1995-2014. Table S3. Incidence rates for chronic morbidities by ten-year period. Table S4. Incidence rates for age-related impairments by ten-year period. (DOCX 35 kb) [file 12963_2017_136_MOESM1_ESM.docx]

**SUPPLEMENTARY MATERIAL**

**Additional Table 1: Incidence rates^1^ of chronic morbidities by age group, 1995-2014**

|  | **Number of events** | **Person years (thousands)** | **Incidence rate (per thousand)** |
| --- | --- | --- | --- |
| **CHD**  80-84  85-89  90-94  95-99  100+ | 9,646  7,937  4,591  1,353  157 | 647.5  536.2  304.9  100.4  18.0 | 14.9 (14.6 to 15.2)  14.8 (14.5 to 15.1)  15.1 (14.6 to 15.5)  13.5 (12.8 to 14.2)  8.7 (7.5 to 10.2) |
| **Stroke**  80-84  85-89  90-94  95-99  100+ | 6,697  7,620  5,405  1,921  277 | 777.2  639.9  354.8  112.6  19.6 | 8.6 (8.4 to 8.8)  11.9 (11.7 to 12.2)  15.2 (14.8 to 15.6)  17.1 (16.3 to 17.8)  14.2 (12.6 to 15.9) |
| **Cancer**  80-84  85-89  90-94  95-99  100+ | 14,553  12,938  6,905  1,820  182 | 691.0  559.8  310.2  99.8  17.9 | 21.1 (20.7 to 21.4)  23.1 (22.7 to 23.5)  22.3 (21.7 to 22.8)  18.2 (17.4 to 19.1)  10.2 (8.8 to 11.7) |
| **COPD**  80-84  85-89  90-94  95-99  100+ | 7,920  5,542  2,498  626  75 | 673.4  565.0  324.3  106.1  19.0 | 11.8 (11.5 to 12.0)  9.8 (9.5 to 10.1)  7.7 (7.4 to 8.0)  5.9 (5.5 to 6.4)  4.0 (3.2 to 5.0) |
| **DM**  80-84  85-89  90-94  95-99  100+ | 5,714  2,742  655  53  0 | 747.8  647.4  376.4  122.1  21.3 | 7.6 (7.4 to 7.8)  4.2 (4.1 to 4.4)  1.7 (1.6 to 1.9)  0.4 (0.3 to 0.6)  0 |

^1^Adjusted for time or year of turning 80 (80-84 age group); year of turning 85 (85-89 age group); year of turning 90 (90-94 age group); year of turning 95 (95-99 age group); year of turning 100 (100+ age group).

**Additional Table 2: Incidence rates^1^ of age-related impairments by age group, 1995-2014**

|  | **Number of events** | **Person years (thousands)** | **Incidence rate (per thousand)** |
| --- | --- | --- | --- |
| **Dementia**  80-84  85-89  90-94  95-99  100+ | 8,044  12,074  8,660  2,898  252 | 803.6  652.1  353.1  109.0  19.3 | 10.0 (9.8 to 10.2)  18.5 (18.2 to 18.8)  24.5 (24.0 to 25.0)  26.6 (25.6 to 27.6)  13.0 (11.5 to 14.7) |
| **Cognitive/memory**  80-84  85-89  90-94  95-99  100+ | 8,382  9,699  5,611  1,503  117 | 797.6  654.5  363.0  115.6  20.4 | 10.5 (10.3 to 10.7)  14.8 (14.5 to 15.1)  15.5 (15.1 to 15.9)  13.0 (12.4 to 13.7)  5.7 (4.8 to 6.9) |
| **MSK pain**  80-84  85-89  90-94  95-99  100+ | 27,541  21,397  11,139  3,236  429 | 489.2  396.0  226.4  77.4  14.9 | 56.3 (55.6 to 56.9)  54.0 (53.3 to 54.8)  49.2 (48.3 to 50.1)  41.8 (40.4 to 43.3)  28.7 (26.1 to 31.6) |
| **Falls**  80-84  85-89  90-94  95-99  100+ | 20,322  24,314  17,018  5,779  730 | 719.9  558.3  287.6  85.7  15.3 | 28.2 (27.8 to 28.6)  43.6 (43.0 to 44.1)  59.2 (58.3 to 60.1)  67.4 (65.7 to 69.2)  47.8 (44.5 to 51.4) |
| **Fractures**  80-84  85-89  90-94  95-99  100+ | 11,948  13,454  9,250  3,299  410 | 655.7  522.3  275.5  82.7  14.9 | 18.2 (17.9 to 18.6)  25.8 (25.3 to 26.2)  33.6 (32.9 to 34.3)  39.9 (38.6 to 41.3)  27.5 (25.0 to 30.3) |
| **Hearing impairment**  80-84  85-89  90-94  95-99  100+ | 18,241  16,963  9,448  2,628  277 | 651.6  505.1  265.4  81.7  18.3 | 28.0 (27.6 to 28.4)  33.6 (33.1 to 34.1)  35.6 (34.9 to 36.3)  32.2 (31.0 to 33.4)  18.3 (16.3 to 20.6) |
| **Visual impairment**  80-84  85-89  90-94  95-99  100+ | 8,373  8,267  4,950  1,314  136 | 754.4  609.6  329.8  102.1  18.0 | 11.1 (10.9 to 11.3)  13.6 (13.3 to 13.9)  15.0 (14.6 to 15.4)  12.9 (12.2 to 13.6)  7.6 (6.4 to 8.9) |

^1^Adjusted for year of turning 80 (80-84 age group); year of turning 85 (85-89 age group); year of turning 90 (90-94 age group); year of turning 95 (95-99 age group); year of turning 100 (100+ age group).

**Additional Table 3: Incidence rates for chronic morbidities by ten-year period.**

|  | **Period A (1995-2004)** | | | **Period B (2005-2014)** | | |  |
| --- | --- | --- | --- | --- | --- | --- | --- |
|  | **Number of events** | **Person years (thousands)** | **Incidence rate (per thousand)** | **Number of events** | **Person years (thousands)** | **Incidence rate (per thousand)** | **Period B hazard ratio^1^** |
| **CHD**  80-84  85-89  90-94  95-99  100+ | 5,015  3,642  2,026  570  60 | 288.7  203.3  112.2  35.8  5.7 | 17.4 (16.9 to 17.9)  17.9 (17.3 to 18.5)  18.1 (17.3 to 18.9)  15.9 (14.7 to 17.3)  10.5 (8.1 to 13.5) | 3,326  3,452  2,196  689  86 | 305.5  299.1  176.8  60.4  11.7 | 10.9 (10.5 to 11.3)  11.5 (11.2 to 11.9)  12.4 (11.9 to 13.0)  11.4 (10.6 to 12.3)  7.3 (5.9 to 9.1) | 0.63* (0.60 to 0.66)  0.64* (0.61 to 0.67)  0.68* (0.64 to 0.72)  0.72* (0.64 to 0.81)  0.77 (0.55 to 1.07) |
| **Stroke**  80-84  85-89  90-94  95-99  100+ | 3,123  3,177  2,283  853  111 | 344.3  239.1  128.1  39.2  6.1 | 9.1 (8.8 to 9.4)  13.3 (12.8 to 13.8)  17.8 (17.1 to 18.6)  21.7 (20.3 to 23.2)  18.3 (15.2 to 22.1) | 2,984  3,854  2,740  946  150 | 372.6  363.3  209.5  68.9  12.9 | 8.0 (7.7 to 8.3)  10.6 (10.3 to 10.9)  13.1 (12.6 to 13.6)  13.7 (12.9 to 14.6)  11.6 (9.9 to 13.6) | 0.85* (0.80 to 0.89)  0.78* (0.75 to 0.82)  0.73* (0.69 to 0.77)  0.64* (0.58 to 0.70)  0.69* (0.54 to 0.88) |
| **Cancer**  80-84  85-89  90-94  95-99  100+ | 5,654  4,171  2,281  589  45 | 313.5  217.5  117.3  36.5  5.8 | 18.0 (17.6 to 18.5)  19.2 (18.6 to 19.8)  19.4 (18.7 to 20.3)  16.2 (14.9 to 17.5)  7.8 (5.8 to 10.4) | 7,918  8,090  4,285  1,160  125 | 320.6  306.7  176.2  59.0  11.5 | 24.7 (24.2 to 25.2)  26.4 (25.8 to 27.0)  24.3 (23.6 to 25.1)  19.7 (18.6 to 20.8)  10.8 (9.1 to 12.9) | 1.31* (1.26 to 1.36)  1.33* (1.28 to 1.39)  1.23* (1.16 to 1.29)  1.25* (1.13 to 1.38)  1.58* (1.12 to 2.23) |
| **COPD**  80-84  85-89  90-94  95-99  100+ | 3,367  2,203  1,045  292  35 | 297.9  210.7  116.4  36.7  5.8 | 11.3 (10.9 to 11.7)  10.5 (10.0 to 10.9)  9.0 (8.4 to 9.5)  8.0 (7.1 to 8.9)  6.0 (4.3 to 8.4) | 2,835  2,317  961  215  27 | 322.4  321.9  192.1  65.3  12.6 | 8.8 (8.5 to 9.1)  7.2 (6.9 to 7.5)  5.0 (4.7 to 5.3)  3.3 (2.9 to 3.8)  2.1 (1.5 to 3.1) | 0.80* (0.76 to 0.85)  0.70* (0.66 to 0.74)  0.56* (0.51 to 0.61)  0.43* (0.36 to 0.52)  0.39* (0.24 to 0.65) |
| **DM**  80-84  85-89  90-94  95-99  100+ | 2,228  657  90  0  0 | 341.6  250.0  138.2  42.5  6.4 | 6.5 (6.3 to 6.8)  2.6 (2.4 to 2.8)  0.7 (0.5 to 0.8)  0  0 | 3,348  2,085  565  53  0 | 344.8  357.9  219.9  74.9  14.2 | 9.7 (9.4 to 10.0)  5.8 (5.6 to 6.1)  2.6 (2.4 to 2.8)  0.7 (0.5 to 0.9)  0 | 1.46* (1.38 to 1.54)  2.24* (2.05 to 2.45)  4.17* (3.33 to 5.21)  --  -- |

^1^Adjusted for sex and time.

**Additional Table 4: Incidence rates for age-related impairments by ten-year period.**

|  | **Period A (1995-2004)** | | | **Period B (2005-2014)** | | |  |
| --- | --- | --- | --- | --- | --- | --- | --- |
|  | **Number of events** | **Person years (thousands)** | **Incidence rate (per thousand)** | **Number of events** | **Person years (thousands)** | **Incidence rate (per thousand)** | **Period B hazard ratio^1^** |
| **Dementia**  80-84  85-89  90-94  95-99  100+ | 2,490  3,344  2,425  751  56 | 357.4  246.7  130.3  39.2  6.1 | 7.0 (6.7 to 7.2)  13.6 (13.1 to 14.0)  18.6 (17.9 to 19.4)  19.2 (17.9 to 20.6)  9.1 (7.0 to 11.9) | 5,215  8,356  5,969  2,065  190 | 384.0  367.0  205.4  65.4  12.6 | 13.6 (13.2 to 14.0)  22.8 (22.3 to 23.3)  29.1 (28.3 to 29.8)  31.6 (30.3 to 33.0)  15.1 (13.1 to 17.4) | 1.89* (1.80 to 1.98)  1.67* (1.60 to 1.74)  1.58* (1.51 to 1.66)  1.70* (1.56 to 1.85)  1.93* (1.43 to 2.60) |
| **Cognitive/memory**  80-84  85-89  90-94  95-99  100+ | 2,320  2,107  1,127  277  20 | 358.0  250.8  135.4  41.5  6.3 | 6.5 (6.2 to 6.8)  8.4 (8.1 to 8.8)  8.3 (7.9 to 8.8)  6.7 (5.9 to 7.5)  3.2 (2.0 to 4.9) | 5,919  7,498  4,442  1,214  96 | 377.0  364.4  209.4  69.3  13.4 | 15.7 (15.3 to 16.1)  20.6 (20.1 to 21.0)  21.2 (20.6 to 21.8)  17.5 (16.6 to 18.5)  7.1 (5.8 to 8.7) | 2.29* (2.18 to 2.40)  2.41* (2.30 to 2.54)  2.55* (2.39 to 2.73)  2.68* (2.35 to 3.06)  2.61* (1.61 to 4.23) |
| **MSK pain**  80-84  85-89  90-94  95-99  100+ | 11,818  8,018  4,073  1,084  106 | 237.8  168.3  94.0  30.9  5.1 | 49.7 (48.8 to 50.6)  47.6 (46.6 to 48.7)  43.3 (42.0 to 44.7)  35.1 (33.1 to 37.3)  20.7 (17.1 to 25.0) | 12,120  11,078  6,106  1,934  288 | 201.2  195.9  117.5  42.5  9.3 | 60.2 (59.2 to 61.3)  56.5 (55.5 to 57.6)  52.0 (50.7 to 53.3)  45.5 (43.5 to 47.6)  31.0 (27.6 to 34.8) | 1.24* (1.20 to 1.27)  1.21* (1.18 to 1.25)  1.22* (1.17 to 1.27)  1.32* (1.23 to 1.43)  1.75* (1.40 to 2.19) |
| **Falls**  80-84  85-89  90-94  95-99  100+ | 8,437  8,758  5,887  1,913  220 | 325.4  216.1  109.7  32.1  5.1 | 25.9 (25.4 to 26.5)  40.5 (39.7 to 41.4)  53.7 (52.3 to 55.1)  59.6 (57.0 to 62.3)  43.3 (38.0 to 49.5) | 10,201  13,857  10,062  3,539  478 | 335.4  306.4  162.2  49.7  9.7 | 30.4 (29.8 to 31.0)  45.2 (44.5 to 46.0)  62.0 (60.8 to 63.3)  71.1 (68.8 to 73.5)  49.5 (45.3 to 54.2) | 1.15* (1.11 to 1.18)  1.10* (1.07 to 1.13)  1.15* (1.11 to 1.18)  1.20* (1.14 to 1.27)  1.32* (1.12 to 1.55) |
| **Fractures**  80-84  85-89  90-94  95-99  100+ | 4,825  4,488  3,059  1,037  120 | 297.6  203.2  105.6  31.6  5.2 | 16.2 (15.8 to 16.7)  22.1 (21.4 to 22.7)  29.0 (28.0 to 30.0)  32.9 (30.9 to 34.9)  23.2 (19.4 to 27.8) | 6,223  8,209  5,716  2,131  271 | 302.6  284.9  154.4  47.1  9.2 | 20.6 (20.1 to 21.1)  28.8 (28.2 to 29.4)  37.0 (36.1 to 38.0)  45.2 (43.3 to 48.2)  29.4 (26.1 to 33.2) | 1.31* (1.26 to 1.36)  1.35* (1.30 to 1.40)  1.32* (1.26 to 1.38)  1.40* (1.30 to 1.51)  1.49* (1.20 to 1.85) |
| **Hearing**  80-84  85-89  90-94  95-99  100+ | 7,807  6,222  3,358  871  70 | 298.8  201.3  104.8  31.9  5.1 | 26.1 (25.6 to 26.7)  30.9 (30.2 to 31.7)  32.0 (31.0 to 33.1)  27.3 (25.6 to 29.2)  13.6 (10.8 to 17.2) | 9,133  9,774  5,652  1,660  191 | 296.1  269.1  144.8  45.8  9.4 | 30.9 (30.2 to 31.6)  36.3 (35.6 to 37.0)  39.0 (38.0 to 40.1)  36.2 (34.5 to 38.0)  20.2 (17.6 to 23.3) | 1.16* (1.12 to 1.20)  1.17* (1.13 to 1.21)  1.23* (1.18 to 1.28)  1.36* (1.25 to 1.48)  1.76* (1.34 to 2.32) |
| **Visual**  80-84  85-89  90-94  95-99  100+ | 4,175  3,612  1,999  482  58 | 332.0  226.6  119.4  36.2  5.7 | 12.6 (12.2 to 13.0)  15.9 (15.4 to 16.5)  16.7 (16.0 to 17.5)  13.3 (12.2 to 14.6)  10.2 (7.9 to 13.2) | 3,185  3,869  2,616  743  77 | 362.6  346.2  193.5  61.6  11.7 | 8.8 (8.5 to 9.1)  11.2 (10.8 to 11.5)  13.5 (13.0 to 14.0)  12.1 (11.2 to 13.0)  6.6 (5.3 to 8.2) | 0.69* (0.65 to 0.72)  0.70* (0.67 to 0.73)  0.81* (0.77 to 0.86)  0.92 (0.82 to 1.03)  0.75 (0.53 to 1.06) |

^1^Adjusted for sex and time
